# Transcriptomic analysis of *Bifidobacterium longum* subsp. *longum* BBMN68 in response to oxidative shock

**DOI:** 10.1038/s41598-018-35286-7

**Published:** 2018-11-20

**Authors:** Fanglei Zuo, Rui Yu, Man Xiao, Gul Bahar Khaskheli, Xiaofei Sun, Huiqin Ma, Fazheng Ren, Bing Zhang, Shangwu Chen

**Affiliations:** 1 Beijing Advanced Innovation Center for Food Nutrition and Human Health, College of Food Science and Nutritional Engineering, China Agricultural University, Beijing, 100083 P. R. China; 2 Key Laboratory of Functional Dairy, Department of Food Science and Engineering, College of Food Science and Nutritional Engineering, China Agricultural University, Beijing, 100083 P. R. China; 3 Department of Fruit Tree Sciences, College of Horticulture, China Agricultural University, Beijing, 100193 P. R. China; 4 Core Genomic Facility, Beijing Institute of Genomics, Chinese Academy of Sciences, Beijing, 100101 P. R. China; 5 Present Address: Department of Molecular Biosciences, The Wenner-Gren Institute, Stockholm University, SE-10691 Stockholm, Sweden

## Abstract

*Bifidobacterium longum* strain BBMN68 is sensitive to low concentrations of oxygen. A transcriptomic study was performed to identify candidate genes for *B. longum* BBMN68’s response to oxygen treatment (3%, v/v). Expression of genes and pathways of *B. longum* BBMN68 involved in nucleotide metabolism, amino acid transport, protein turnover and chaperones increased, and that of carbohydrate metabolism, translation and biogenesis decreased to adapt to the oxidative stress. Notably, expression of two classes of ribonucleotide reductase (RNR), which are important for deoxyribonucleotide biosynthesis, was rapidly and persistently induced. First, the class Ib RNR NrdHIEF was immediately upregulated after 5 min oxygen exposure, followed by the class III RNR NrdDG, which was upregulated after 20 min of exposure. The upregulated expression of branched-chain amino acids and tetrahydrofolate biosynthesis-related genes occurred in bifidobacteria in response to oxidative stress. These change toward to compensate for DNA and protein damaged by reactive oxygen species (ROS). In addition, oxidative stress resulted in improved *B. longum* BBMN68 cell hydrophobicity and autoaggregation. These results provide a rich resource for our understanding of the response mechanisms to oxidative stress in bifidobacteria.

## Introduction

Bifidobacteria are Gram-positive, heterofermentative, non-motile, non-spore-forming, anaerobic bacteria that are mainly found in gastrointestinal tract (GIT) of mammals^1,2^. Some bifidobacteria are considered to be probiotic due to their contribution to the maintenance of gastrointestinal health^2,3^. Thus, bifidobacteria are incorporated into many food products, such as yogurt, fermented milk and dietary supplements^3^. However, the efficacy of their probiotic properties can be compromised by their high sensitivity to environmental challenges, especially oxygen-induced oxidative stress^4,5^; nevertheless, some strains can tolerate 5% to 21% (v/v) oxygen^6,7^. Incomplete reduction of oxygen forms reactive oxygen species (ROS), which can cause deleterious effects, including protein misfolding and aggregation, DNA damage and lipid peroxidation^8^.

Enzymes such as NADH oxidase, NADH peroxidase, catalase and superoxide dismutase play key roles in removing ROS in many anaerobic microorganisms^9,10^. In the more than 50 published genome sequences of bifidobacteria^11^, no genes encoding NADH peroxidase, catalase or superoxide dismutase have been annotated (with the exception of *Bifidobacterium asteroides*, which contains a heme-catalase gene^12^). Previous study suggested that alkyl hydroperoxide reductase is probably the primary scavenger of the endogenous hydrogen peroxide (H_2_O_2_) generated during aerobic cultivation of *Bifidobacterium longum*^13^. NADH oxidase and oxygen-dependent coproporphyrinogen III oxidase are involved in detoxifying molecular oxygen and/or H_2_O_2_ in *Bifidobacterium animalis*^14^. Thus, alkyl hydroperoxide reductase, thioredoxin reductase and NADH oxidase are critical in bifidobacteria’s response to oxidative stress^2,15^, as confirmed by proteomic and transcriptomic analyses^14,16–18^. On the other hand, bifidobacteria employ a particular set of proteins, mainly molecular chaperones and proteases, to protect the cells from damage caused by the accumulation of unfolded and/or misfolded proteins. These chaperones and proteases play key roles in several post-translational events to prevent protein denaturation, aggregation and misfolding caused by stresses, such as oxidative stress^19,20^. Induction and assembly of the stress-response system are controlled by a set of complex transcription factors. A report on oxidative responses in *Bifidobacterium breve* showed that HspR, LexA, HrcA, and Crp regulon are involved in the responses to oxygen, H_2_O_2_, and peroxides caused oxidative stress^19^. Among them, RecA–LexA is the major regulator of the SOS response in bacteria induced by DNA damage^21^, and HspR regulates *dnak*, *clpB*, and *clgR*, which are involved in heat, osmosis, and solvent stress responses, respectively^22^.

Despite physiological and biochemical analyses carried out in the last decade and the accumulation of ‘omics’ studies in recent years providing information on oxidative stress responses in bifidobacteria^6,14–18,23^, the global gene-transcription profile in response to oxygen stress in bifidobacteria has not been well elucidated. *B. longum* subsp. *longum* BBMN68 is a gut-inhabiting strain isolated from a healthy centenarian which has a number of probiotic properties^24–26^. It is very sensitive to low and residual oxygen, and headspace contact with 3% to 6% oxygen yields severe to sublethal growth inhibition^16^. In the present study, next-generation RNA-sequencing (RNA-Seq) analysis and validation of physiological characteristics were employed to study the oxidative stress response and resistance mechanism in *B. longum* strain BBMN68.

## Materials and Methods

### Bacterial strains and growth conditions

*B. longum* subsp. *longum* strain BBMN68^27^ was cultivated under standard anaerobic conditions in de Man Rogosa Sharpe (MRS) broth (Oxoid) with 0.05% (w/v) L-cysteine HCl (MRSC) at 37 °C in Hungate tubes or infusion vials (300 ml capacity) purged with a gas mixture of 10% (v/v) H_2_, 10% CO_2_, and 80% N_2_, unless otherwise noted^16^.

### Oxygen treatment of *B. longum* BBMN68 culture

Overnight *B. longum* BBMN68 culture was inoculated (1%, v/v) by syringe into injection vials containing 100 ml pre-warmed MRS medium, and the culture was grown at 37 °C. When growth reached the exponential phase (optical density at 600 nm [OD_600_] = 0.5, after about 6 h cultivation), 3% (v/v) oxygen was established in the injection vial headspace by previously reported methods^16^. After the modulation of the headspace gas component, injection vials were incubated at 37 °C with gentle horizontal shaking (100 rpm). Samples used for RNA extraction were collected from six biological replicates after 30 min and 60 min of oxygen treatment by centrifugation at 8,000 × *g* for 5 min at 4 °C. Culture collected prior to treatment was used as a control.

### RNA extraction, sequencing and annotation

The Applied Biosystems (AB) SOLiD^TM^ 4.0 System Sequencing Analyzer was used for the RNA-Seq analyses. Total RNA was isolated from 10 ml bacterial cells (about 1 × 10^8^ CFU ml^−1^) subjected to the different treatments using TRIzol reagent (Invitrogen, Cat. no. 15596026) according to the manufacturer’s instructions. The mRNA was enriched using a Ribo-minus Kit (Invitrogen, Cat. no. 1083708) that depletes rRNA. A mRNA-Seq library was prepared with the total RNA-Seq Kit (AB) according to the manufacturer’s protocol. cDNA in the 150–200-bp range was selected with Novex precast gel products (Invitrogen, Cat. no. NP0322BOX), amplified by 15 PCR cycles and cleaned with PureLink PCR Micro Kit (Invitrogen, Cat. no. K310250). All sequenced reads were aligned to *B. longum* subsp. *longum* BBMN68 (NC_014656.1) using AB’s SOLiD Corona_lite_v4.2 software. We used a recursive strategy to improve the read-mapping ratio: 50mer reads were first mapped to the genome with a tolerance of five mismatches; the reads that failed to be mapped were progressively trimmed—five bases at a time from the 3′ end—and then mapped to the genome again until a match was found (unless the read was trimmed to less than 30 bases). All of these uniquely mapped reads were used to calculate the gene-expression level in RPKM (reads per kilobase of exon per million mapped sequenced reads). We identified differentially expressed genes from the different samples using the R package DEGseq (http://waprna.big.ac.cn/rnaseq/function/degseq.jsp) with statistically significant level set at *P* < 0.001. The analyzed transcriptomic data were submitted to the Gene Expression Omnibus (GEO) database (http://www.ncbi.nlm.nih.gov/geo/) with accession number GSE65320.

### Real-time quantitative PCR (RT-qPCR) analysis

Reverse transcription was carried out on the total RNA extracted from the treatment and control cultures with M-MLV Reverse Transcriptase (Promega), using 2 µg DNase I-digested total RNA as the template. The absence of residual DNA in the total RNA digested by DNase I was confirmed by PCR. Specific primers for each gene (Table 1) were designed using Primer Premier 5 software. RT-qPCR was performed using SYBR^®^ Premix Ex Taq^TM^ (Takara) and optimized primer concentrations in a LightCycler^®^ 96 Real-Time PCR system (Roche), with cycling and detection of 95 °C for 10 s and 60 °C for 30 s (40 cycles). Gene expression was normalized by the ΔΔC_T_ method^28^, using 16S rRNA as the reference gene in the calculations^14,16^. The experiment was performed in triplicate and the average results are reported.

**Table 1:** Target gene oligonucleotide primers for RT-PCR.

Gene (Locus tag)	Primer sequence (5′ → 3′)		Size of product (bp)
	Forward	Reverse	
*sufB1 (BBMN68_611)*	ACGACGGTGACGCACGACT	AGATGCCGAGCATGTTGAGGT	243
*glycerate kinase (BBMN68_585)*	GCCCTCGGCGTTCGTCTTCT	CAATGTGGCGACATCATCTTTGGA	225
*grxC2 (BBMN68_1397)*	GCAGTGCGATGCCACCAAG	CAGGAGTTGTCCGGCGTGAT	147
*tatC (BBMN68_1285)*	GGAGCCGGACTGGCATGGTATCT	CGTTGCGAGACGCCACTGCTT	228
*hcaD (BBMN68_1524)*	ACGCCAGAACCCTCACCTACC	CCGATCACCACTGCCGACTT	217
*16 S rRNA (BBMN68_rRNA7)*	CGTAGGGTGCAAGCGTTATC	GCCTTCGCCATTGGTGTT	197
*nrdI (BBMN68_1398)*	GGATGCCGTTTGCAGGAC	TCGTTGAGGAAGCGTTTGAC	164
*nrdE (BBMN68_1399)*	CCTGCCGCTCGACAATACT	CTTGAACGCACCAAGGAAAG	334
*nrdF (BBMN68_1401)*	CCCTGCTTGACACCATCC	AACTCGTTGTTCTCGCTCC	199
*nrdD (BBMN68_1785)*	TGCGGTCAAGTCTGCTTTC	CGAGCCACATCGTACAGGT	189
*nrdG (BBMN68_1786)*	TCTTGCCAACGATCCGAAAG	CCGCCAAGGAACGTAATGC	267
*nrdR (BBMN68_197)*	GGAGCCATTCAGTAGAGAC	TCCAGACCTGCAAAGTTC	240

### Autoaggregation and hydrophobicity assay

*B. longum* BBMN68 cells grown in MRSC or MRS for 6 h (exponential phase) were harvested and resuspended in phosphate buffer (pH 6.8) to yield an OD_600_ of 1.0. For the autoaggregation assay, the cell suspension was incubated anaerobically at 37 °C for 3 h and 6 h, and then 0.1 ml of the upper suspension was gently transferred to another tube with 1.9 ml of phosphate buffer and OD_600_ was measured. The percentage of autoaggregation was expressed as (1 − OD_600_ of the upper suspension/OD_600_ of the total bacterial suspension) × 100%^29^. To determine the hydrophobicity of the bifidobacterial cells, 0.6 ml xylene was added to 3 ml of cell suspension and vortexed for 120 s. The aqueous phase was removed after 1 h of incubation at room temperature and its absorbance at 600 nm was measured. Cell-surface hydrophobicity was calculated as (1 - OD_600_ of the aqueous phase suspension/OD_600_ of the total bacterial suspension) × 100%^30,31^.

### Statistical analysis for RT-qPCR and physiological assays results

All of the RT-qPCR and physiological assay data from three independent experiments were analyzed by two-tailed Student’s *t* test. All analyses were performed using Microsoft Office Excel 2007. Values of *P* < 0.05 were considered significant.

## Results and Discussion

### Global transcriptomic analysis of the oxygen response in *B. longum* BBMN68

**Figure 1:**
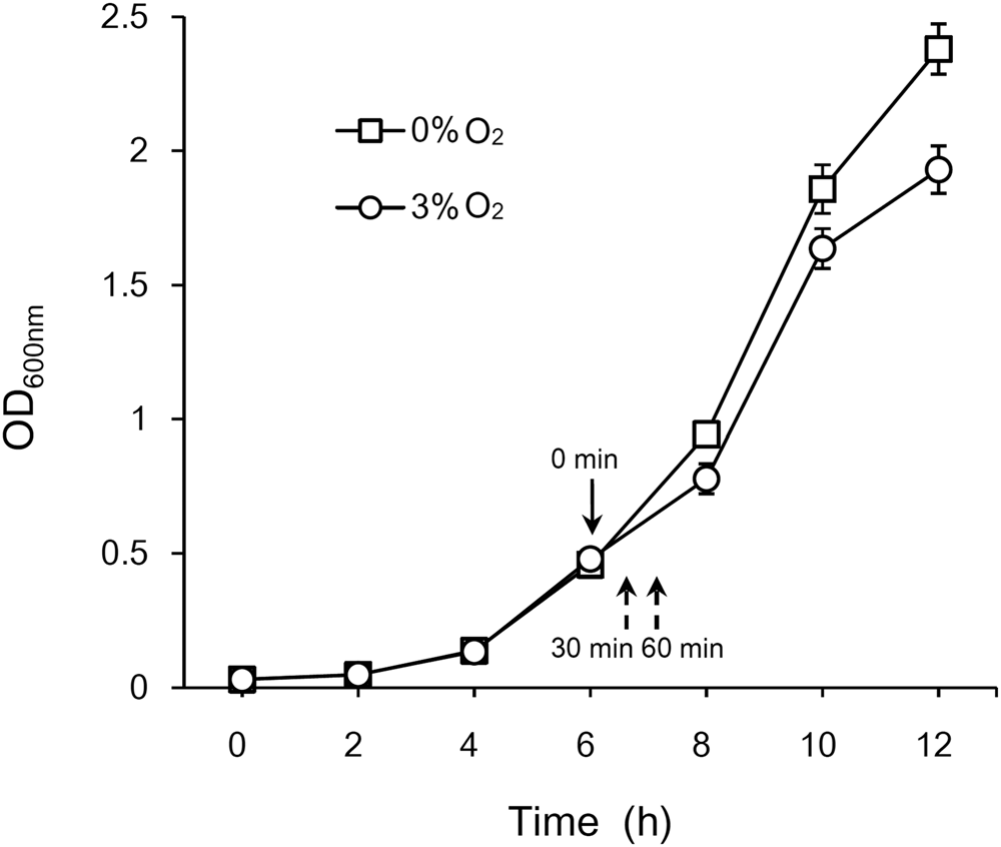
Growth of *B. longum* BBMN68 in MRS with or without 3% (v/v) oxygen challenge. Samples were collected from the time points indicated by arrows.

A previous study, using a proteomic approach to analyze changes in the cellular protein profiles of BBMN68 exposed to an inhibitory to sublethal concentration of oxygen (3%, v/v), revealed some key proteins involved in the response of BBMN68 to oxygen^16^. To further understand the mechanism of bifidobacteria’s response to oxidative stress, BBMN68 cells were treated with 3% oxygen and global transcriptional changes were analyzed by SOLiD 4.0 RNA-Seq. A total of 17,539,582, 23,696,766 and 20,913,996 uniquely mapped reads were obtained for the oxygen-challenged samples harvested at two time points (30 and 60 min) after oxygen delivery, and a reference sample taken prior to oxygen delivery (control, 0 min), respectively (Fig. 1). After filtering, the number of effective reads mapped to the genome of BBMN68 was 13,300,802, 18,504,526 and 14,833,647, respectively. Genes that were significantly differentially expressed (based on a fold change of at least two [log_2_ ratio <−1 or >1] and a t-test *P*-value < 0.001) in response to oxygen were sorted: expression of 99 genes was downregulated and of 241 genes upregulated after 30 min, and expression of 218 genes was downregulated, 217 upregulated after 60 min of oxygen exposure compared to controls (Tables S1 and S2); expression of 70 genes was downregulated, and 124 upregulated at both time points (Tables S2). Some upregulated genes encoding proteins also induced at protein level by proteomics study, such as AhpC, NrdA, Eno^16^.

**Figure 2:**
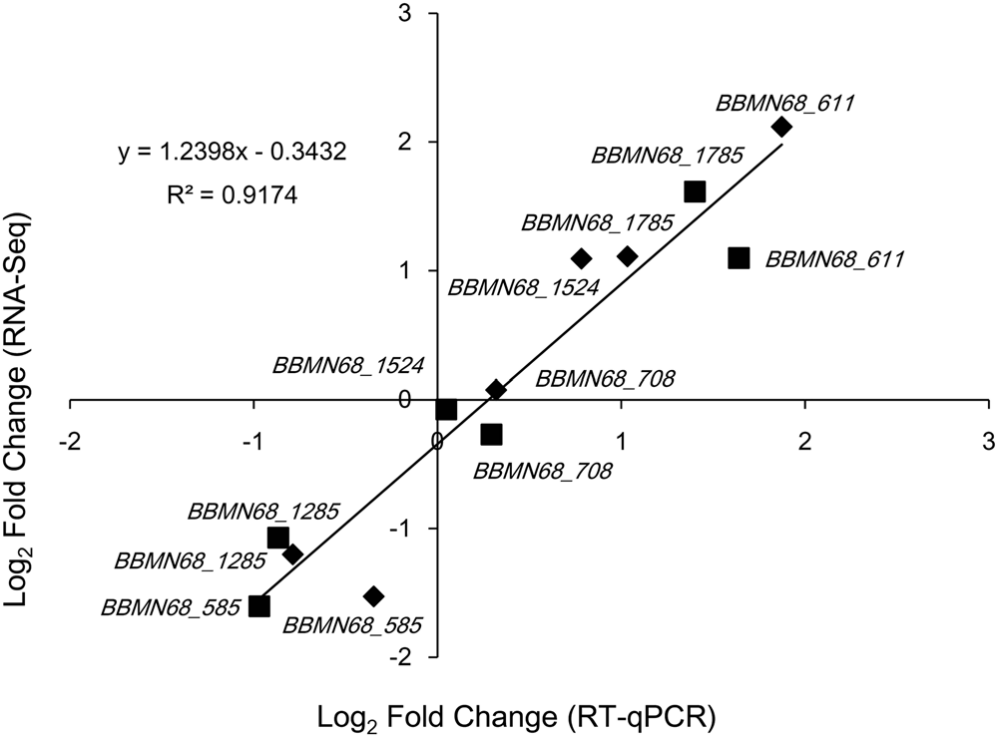
RT-qPCR validation of the RNA-Seq transcriptomic data. Chart shows correlation of fold changes for six genes’ expression from *B. longum* BBMN68 cells after 30 min (squares) or 60 min (diamonds) exposure to 3% (v/v) oxygen, as derived from the transcriptomic analysis and RT-qPCR. The best fit is shown along with the calculated equation and *r*^2^ value.

**Figure 3:**
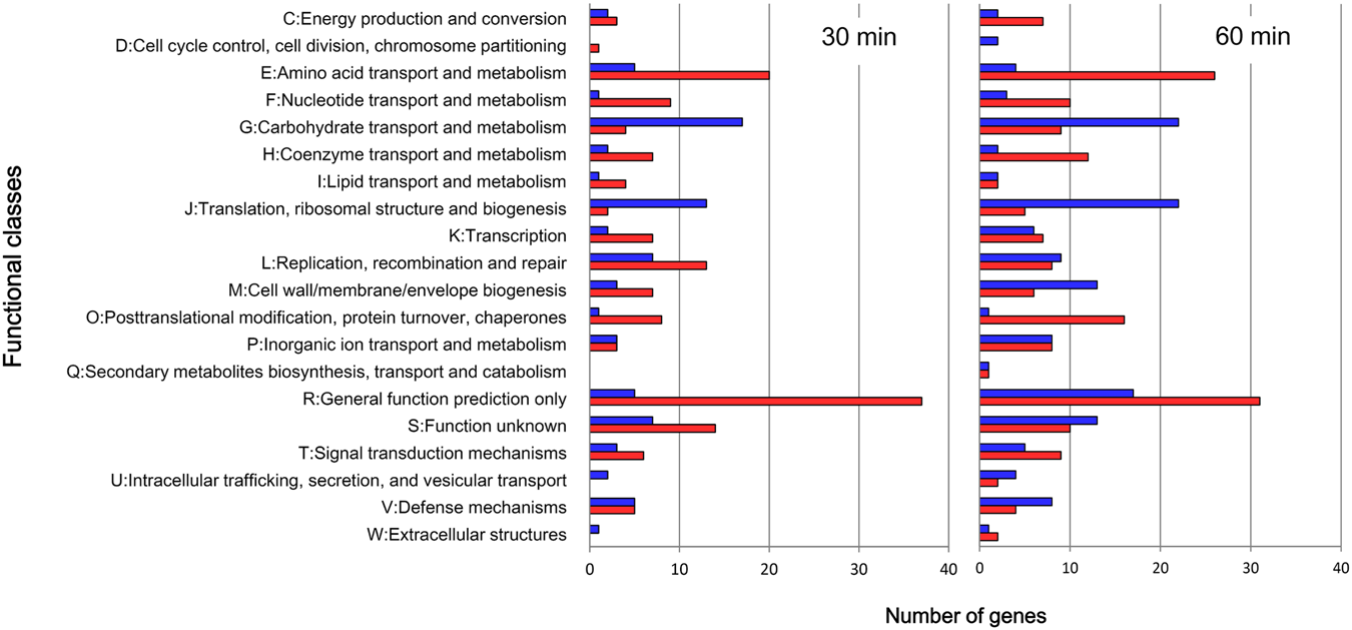
Relative abundance of transcripts assigned to COG functional categories. Functional classification of genes with statistically significant increase (red bar) or decrease (blue bar) in mRNA level after 30 min and 60 min exposure to 3% (v/v) oxygen compared to controls.

Real-time quantitative PCR (RT-qPCR) analysis of six different genes was performed to validate the transcriptomic data. A strong positive correlation (*r*^2^ = 0.92) was found between the fold-change in gene induction or repression obtained from the transcriptomic data and the values determined by RT-qPCR (Fig. 2), suggesting agreement between the two platforms. The differentially expressed genes were grouped into functional categories according to the Clusters of Orthologous Groups (COG) classification system^32^. COG categories E (amino acid transport and metabolism), R (general function prediction), and O (post-translational modification, protein turnover, chaperones) had a high number of upregulated genes after both 30 min and 60 min oxygen treatment compared to controls (Fig. 3). In addition, many genes in categories F (nucleotide transport and metabolism), H (coenzyme metabolism), T (signal-transduction mechanisms) and L (DNA replication, recombination and repair) were also upregulated, whereas most of the genes belonging to categories G (carbohydrate transport and metabolism) and J (translation, ribosomal structure and biogenesis) were downregulated at both time points after oxygen exposure compared to controls (Fig. 3).

The results revealed that *B. longum* BBMN68 cells employ complex defense and adaptation mechanisms to counteract oxygen-driven stresses, including oxygen reduction and ROS detoxification, repair of damaged biomacromolecules, and adaptive modulation of several metabolic processes.

### Detoxification and redox homeostasis

Thioredoxin and glutaredoxin make up the thioredoxin- and glutaredoxin-dependent reduction systems in *Escherichia coli* and many other bacteria, and are responsible for maintaining a reduced environment in the cell cytosol^33^. However, *B. longum* BBMN68 has an incomplete glutaredoxin system which lacks any detectable genes for glutathione peroxidase (GPx) or glutathione reductase (GR)^27^. Two genes encoding glutaredoxin, *grxC1* (*BBMN68_125*) and *grxC2* (*BBMN68_1397*), were upregulated in BBMN68 upon exposure to oxygen. In particular, *grxC2*, also known as *nrdH*, was upregulated more than 6-fold after both 30 min and 60 min oxygen exposure (Table 2). A previous study suggested that glutaredoxin encoded by *nrdH* is reduced by thioredoxin reductase rather than glutathione (GSH)^34^. Thus, the thioredoxin-dependent antioxidant system might be the major redox homeostasis system in strain BBMN68, as *trxB1* (*BBMN68_1345*) encoding thioredoxin reductase was highly upregulated, and *BBMN68_991* encoding the corresponding thioredoxin was also upregulated in BBMN68 after 60 min exposure to oxygen (Table 2). Thioredoxin reductase has been found to respond to oxidative stress at both transcriptional and translational levels in bifidobacteria^15,16^. The thioredoxin-dependent reduction system plays an important role in the oxidative stress response by reducing a number of proteins including peroxiredoxins, directly reducing H_2_O_2_, scavenging hydroxyl radicals, quenching singlet oxygen, and maintaining the intracellular thiol-disulfide balance^35^. Because of the most bifidobacterial species lacking genes encoding catalase or superoxide dismutase, introduce catalase and/or superoxide dismutase into bifidobacterial cells could dramatically improve their oxidative stress tolerance. We have demonstrated this hypothesis recently^36^, and the gene encoding catalase has been integrated into the chromosome of bifidobacteria for generating food-grade strain potentially used in food industry (Our unpublished data).

**Table 2:** Genes differentially expressed at the transcriptional level in *B. longum* BBMN68 exposed to 3% (v/v) oxygen.

Proposed function	Gene name	Log_2_ ratio^a^		Locus tag^b^
		30 min vs. Cont	60 min vs. Cont	
Oxidative response (detoxification)				
Thioredoxin reductase	*trxB1*	3.15	4.09	*BBMN68_1345*
Thioredoxin domain-containing protein		NS	1.14	*BBMN68_991*
Alkyl hydroperoxide reductase subunit C	*ahpC2*	1.38	NS	*BBMN68_1346*
Glutaredoxin	*grxC2* (*nrdH*)	2.68	2.69	*BBMN68_1397*
Glutaredoxin	*grxC1*	NS	1.05	*BBMN68_125*
Energy/intermediary metabolism				
Nitroreductase	*nfnB1*	NS	1.14	*BBMN68_86*
Nitroreductase	*nfnB2*	NS	2.22	*BBMN68_1435*
Class I pyridine nucleotide-disulfide oxidoreductase	*ipd2*	1.79	2.18	*BBMN68_1660*
Dihydroorotate dehydrogenase	*pyrD2*	1.26	2.17	*BBMN68_979*
Nucleic acid repair				
DNA helicase II/ATP-dependent DNA helicase PcrA	*uvrD1*	2.27	2.76	*BBMN68_138*
Excinuclease ABC subunit A	*uvrA1*	NS	1.08	*BBMN68_394*
DNA polymerase V	*dinp1*	1.14	2.08	*BBMN68_863*
ADP-ribose pyrophosphatase		1.76	1.53	*BBMN68_240*
DNA-repair protein RecN	*recN*	1.07	1.20	*BBMN68_793*
Nucleoside triphosphate pyrophosphohydrolase	*mutT3*	1.04	1.11	*BBMN68_1517*
Ribonucleoside-triphosphate reductase	*nrdG*	2.67	1.92	*BBMN68_1786*
Ribonucleoside-triphosphate reductase	*nrdD*	1.61	1.11	*BBMN68_1785*
Protein involved in ribonucleotide reduction	*nrdI*	2.06	1.63	*BBMN68_1398*
Ribonucleoside-diphosphate reductase alpha chain	*nrdE*	3.47	3.01	*BBMN68_1399*
Ribonucleoside-diphosphate reductase beta chain	*nrdF*	5.21	4.77	*BBMN68_1401*
Iron-responsive/iron-related (metal metabolism)				
Cysteine desulfurase	*csdB*(*sufS*)	NS	1.78	*BBMN68_609*
Fe–S cluster assembly protein SufB	*sufB2*	NS	1.49	*BBMN68_612*
Fe–S cluster assembly protein SufD	*sufB1*	1.10	2.12	*BBMN68_611*
Iron complex transport system ATP-binding protein	*modF*	NS	1.46	*BBMN68_569*
P-type ATPase	*zntA1*	NS	1.01	*BBMN68_1149*
Protein repair/chaperones				
Heat-shock molecular chaperone	*ibpA*	2.91	2.77	*BBMN68_1305*
Molecular chaperone DnaJ	*dnaJ1*	NS	1.44	*BBMN68_410*
Co-chaperonin HSP10	*groES*	NS	1.94	*BBMN68_1589*
Chaperonin HSP60	*groEL*	NS	1.27	*BBMN68_44*
ATP-dependent Clp proteases; protease subunit ClpB	*clpA2*	NS	1.48	*BBMN68_1510*
Proteases				
ATP-dependent Clp proteases; protease subunit	*clpP1*	NS	1.43	*BBMN68_692*
ATP-dependent Clp proteases; protease subunit	*clpP2*	NS	1.41	*BBMN68_693*
Protease I	*thiJ*	1.81	1.45	*BBMN68_377*
Putative endopeptidase	*pepO*	NS	1.03	*BBMN68_1763*
Leader peptidase (prepilin peptidase)/N-methyltransferase		2.08	2.46	*BBMN68_618*
Glycolysis				
Probable phosphoglycerate mutase	*phoE*	1.88	3.24	*BBMN68_1437*
3-Bisphosphoglycerate-dependent phosphoglycerate mutase	*gpmA*	NS	1.07	*BBMN68_1687*
Aldehyde dehydrogenase (NAD+)	*putA1*	NS	1.08	*BBMN68_872*
Enolase	*eno*	NS	2.11	*BBMN68_771*
Valine, leucine and isoleucine biosynthesis				
2-Isopropylmalate synthase	*leuA*	1.35	1.13	*BBMN68_1222*
3-Isopropylmalate dehydrogenase	*leuB*	1.55	1.42	*BBMN68_984*
3-Isopropylmalate/(R)-2-methylmalate dehydratase large subunit	*leuC*	2.32	2.98	*BBMN68_1521*
3-Isopropylmalate/(R)-2-methylmalate dehydratase small subunit	*leuD*	1.42	1.78	*BBMN68_1522*
Ketol-acid reductoisomerase	*ilvc1*	NS	1.44	*BBMN68_1262*
Ketol-acid reductoisomerase	*ilvc2*	1.55	1.28	*BBMN68_1263*
Branched-chain amino acid aminotransferase	*ilvE*	2.02	1.40	*BBMN68_592*
Branched-chain amino acid transport system substrate-binding protein	*livK*	1.63	1.93	*BBMN68_1747*
Branched-chain amino acid transport system permease protein	*livH*	1.46	1.74	*BBMN68_1748*
Branched-chain amino acid transport system permease protein	*livM*	1.27	1.61	*BBMN68_1749*
Branched-chain amino acid transport system ATP-binding protein	*livG*	1.72	1.76	*BBMN68_1750*
Branched-chain amino acid transport system ATP-binding protein	*livF*	1.33	1.59	*BBMN68_1751*
Carbohydrate transport systems				
Solute-binding protein of ABC transporter system		−2.01	−1.97	*BBMN68_1170*
Sugar ABC transporter ATP-binding protein	*mglA3*	NS	−1.10	*BBMN68_1727*
Putative multiple sugar transport system permease protein	*xylH*	−1.75	−1.59	*BBMN68_1728*
MalE-type ABC sugar transport system periplasmic component		−2.03	−1.98	*BBMN68_217*
MalF-type ABC sugar transport systems permease component		−2.66	−1.62	*BBMN68_218*
MalG-type ABC sugar transport system permease component		−1.74	−1.49	*BBMN68_219*
Transmembrane transport protein		2.06	2.07	*BBMN68_1264*
Transmembrane transporter activity; MFS transporter (putative metabolite:H^**+**^ **symporter)**		1.63	1.52	*BBMN68_157*
Peptide transport				
Peptide/nickel transport system substrate-binding protein	*ddpA1*	1.16	NS	*BBMN68_236*
Peptide/nickel transport system permease protein	*dppB1*	1.29	1.66	*BBMN68_237*
Peptide/nickel transport system ATP-binding protein	*appF1*	1.05	1.06	*BBMN68_239*
Folate biosynthesis				
GTP cyclohydrolase I	*folE*	1.36	1.93	*BBMN68_1717*
Dihydroneopterin aldolase/2-amino-4-hydroxy-6-hydroxymethyldihydro-				
pteridine diphosphokinase	*folB*	NS	1.15	*BBMN68_1719*
Dihydropteroate synthase	*folP*	NS	1.73	*BBMN68_1718*
Dihydrofolate synthase/folylpolyglutamate synthase	*folC*	NS	1.49	*BBMN68_243*
Dihydrofolate reductase	*folA*	NS	1.36	*BBMN68_1698*
Cell wall/membrane/envelope biogenesis				
Cyclopropane-fatty-acyl-phospholipid synthase	*cfa*	1.44	2.27	*BBMN68_1705*
Bile salt hydrolase	*cbaH*	NS	1.83	*BBMN68_536*
Hypothetical protein		−1.49	−2.04	*BBMN68_1491*
Rhamnosyltransferase		−1.86	−1.93	*BBMN68_1492*
Rhamnosyltransferase		NS	−1.21	*BBMN68_1493*
ABC-2 type transport system permease protein	*tagG*	NS	−1.43	*BBMN68_1495*
ABC-2 type transport system ATP-binding protein	*tagH*	NS	−1.29	*BBMN68_1496*
S-layer protein		−1.46	−1.40	*BBMN68_882*
Signal transduction				
Two-component system, OmpR family, response regulator RegX3		1.62	1.14	*BBMN68_1079*
Histidine kinase sensor of two-component system		1.48	1.64	*BBMN68_1678*
Response regulator of two-component system		NS	1.02	*BBMN68_750*
S-ribosylhomocysteine lyase	*luxS*	1.16	1.75	*BBMN68_914*
Transcriptional factors				
Transcriptional regulator of heat shock	*hrcA*	NS	2.26	*BBMN68_409*
LacI-type transcriptional repressor		1.71	1.61	*BBMN68_223*
SOS-response transcriptional repressor	*lexA1*	1.44	1.81	*BBMN68_195*
Leucine-responsive regulatory protein	*irp*	1.50	1.39	*BBMN68_1361*
Atypical LysR-type transcriptional regulator	*lysR*	NS	1.38	*BBMN68_843*
Putative transcriptional regulator		2.44	2.51	*BBMN68_1661*
Putative transcriptional regulator		1.29	NS	*BBMN68_905*
Hypothetical protein				
Hypothetical protein		4.79	4.64	*BBMN68_1400*
Hypothetical protein		4.63	4.00	*BBMN68_582*
Hypothetical protein		2.87	3.49	*BBMN68_105*
Hypothetical protein		1.92	3.02	*BBMN68_248*
Hypothetical protein		1.70	2.71	*BBMN68_519*
Hypothetical protein		1.58	2.39	*BBMN68_520*
Hypothetical protein		1.92	2.20	*BBMN68_1662*

^a^Log_2_ ratio represents the ratio of mRNA transcript levels in oxygen-treated samples (30 min and 60 min) to untreated samples (Cont).

^b^Open reading frame (ORF) ID is as annotated in KEGG (http://www.genome.jp/kegg/kegg2.html).

NS, not statistically significant.

Interestingly, two nitroreductase-homolog genes, *nfnB1* (*BBMN68_86*) and *nfnB2* (*BBMN68_1435*), were markedly induced after 60 min exposure of BBMN68 to oxygen (Table 2). NfnB2 shows 46.9% amino acid identity with NfrA1 from *Bacillus subtilis*^37^. In the latter, NfrA1 plays a dual role that leads to high concentrations of H_2_O_2_ based on its NADH oxidase activity, whereas it can also scavenge H_2_O_2_ and degrade NAD^+^ ^37^. No high homology of NfnB1 with identified proteins in well-studied bacteria has been found. Nitroreductase is involved in the defense against oxidative stress in *Lactococcus lactis*^38^ and *Staphylococcus aureus*^39^. Therefore, the two nitroreductases might protect *B. longum* BBMN68 from oxygen-induced oxidative stress, warranting further investigation.

In *Lactobacillus plantarum*, Mn^2+^ not only replaces superoxide dismutase in scavenging superoxide anions, but it can also scavenge H_2_O_2_^40^. It has been reported that P-type ATPase might be involved in taking up Mn^2+^, which then scavenges superoxide anions in bifidobacteria^41^. In strain BBMN68, expression of the homologous protein-encoding gene *zntA1* (*BBMN68_1149*) was upregulated 2.01-fold after 60 min of oxygen exposure (Table 2). In addition, BBMN68 grew faster in MRS broth supplemented with Mn^2+^ than in the non-supplemented MRS upon exposure to 3% oxygen (Fig. S1A), but it grew normally under anaerobic conditions (Fig. S1B). This result suggested that manganese can protect bifidobacteria from oxidative stress.

### Oxygen induces a multiple stress response in BBMN68

Chaperones and proteases related to several stress conditions were induced in strain BBMN68 in response to oxygen (Fig. 3, Table 2). The transcription of *groEL* (*BBMN68_44*) and *groES* (*BBMN68_1589*) was upregulated after 60 min exposure to oxygen. The GroEL/GroES complex is required for proper protein folding and is frequently involved in responses to heat, low-pH and bile-salt stresses in bifidobacteria^42–45^. Genes encoding other chaperones, such as *BBMN68_410* and *BBMN68_1510* encoding DnaJ and ClpB genes, respectively, were upregulated in strain BBMN68 after 60 min exposure to oxygen (Table 2). ClpB cooperates with DnaK, DnaJ, and GrpE in suppressing protein aggregation; this is a universal phenomenon found in different organisms’ responses to various abiotic stress conditions^46,47^. Note that expression of the gene *BBMN68_1305* encoding the small heat-shock protein (sHsp) IbpA was consecutively induced more than 6-fold after both 30 min and 60 min of oxygen exposure in BBMN68. The *ibpA* homolog *BL0576* is the most rapidly and strongly induced gene in *B. longum* NCC2705’s response to oxidative stress^41^. This result suggested that IbpA is important in preventing protein aggregation and misfolding, representing an early and persistent response to oxidative stress in BBMN68. In addition, several genes encoding proteases and peptidases were upregulated in BBMN68 after 60 min exposure to oxygen, including *clpP1* (*BBMN68_692*), *clpP2* (*BBMN68_693*), *thiJ* (*BBMN68_377*), and *pepO* (*BBMN68_1763*) (Table 2). These proteases and peptidases play a major role in the degradation and turnover of damaged proteins.

Genes involved in the SOS response were also upregulated. The SOS response in bacteria is a global regulatory network for DNA-damage repair, governed by the repressor LexA and inducer RecA^21^. In BBMN68, *lexA* (*BBMN68_195*) expression was upregulated 2.72- and 3.50-fold after 30 and 60 min oxygen exposure, respectively (Table 2). Accordingly, several genes belonging to the *LexA* regulon were also upregulated upon exposure to oxygen (Table 2). Among them, the DNA-repair protein RecN encoded by *BBMN68_793* was upregulated 2.09- and 2.30-fold in BBMN68 upon oxygen exposure for 30 and 60 min, respectively; this protein has also been shown to be regulated by the ferric-uptake regulator (Fur) and to play a role in oxidative-damage protection in *Neisseria gonorrhoeae*^48^. This result suggested that oxygen-induced DNA damage leads to activation of RecA–LexA, which subsequently protects BBMN68 from oxidative stress.

### Effect of oxygen stress on carbohydrate, nucleotide, and amino acid metabolism

Most of the genes involved in carbohydrate transport and metabolism, belonging to COG category G, were downregulated in strain BBMN68 relative to controls, especially after 30 min exposure to oxygen (Fig. 3). An overall transcriptome map presents a clear picture of the proposed carbohydrate metabolism of BBMN68 grown under oxygen stress^49^ (Fig. S2). In general, the expression profiles of genes involved in the glycolysis and pentose phosphate pathways were not significantly modified. However, the expression of genes encoding three enzymes related to utilization of complex carbohydrate sources—enolase (*BBMN68_771*) and two phosphoglycerate mutases (*BBMN68_1437*, *BBMN68_1687*)—was upregulated in BBMN68 after 60 min of oxygen exposure (Table 2). Enolase overproduction in BBMN68’s response to oxygen was also confirmed in our previous proteomics study^16^; these three enzymes fuel the bifid shunt, although expression of genes encoding the key enzymes of that shunt—fructose-6-phosphate phosphoketolase (FPPK, BBMN68_708) and glyceraldehyde 3-phosphate dehydrogenase (Gap, BBMN68_254)—was not significantly induced. Many of the genes encoding proteins in oligosaccharide and disaccharide metabolism were downregulated relative to controls (Table S2), suggesting that polysaccharide utilization is repressed in BBMN68 in response to oxidative stress. On the other hand, the following transport systems genes were heavily downregulated: *BBMN68_1170* encoding a solute-binding protein of the ABC transporter system and predicted to be a putative transporter for oligofructose^50^; *BBMN68_1728* encoding a putative multiple sugar transport system permease protein and suggested to be involved in the transport of multiple sugars with fructose and mannose moieties^50^; *BBMN68_217–219* encoding proteins involved in transporting mannose-containing oligosaccharides^50^ (Table 2). These results corresponded with the repressed polysaccharide and oligosaccharide utilization in BBMN68 under oxygen stress, which has also been detected in BBMN68 in response to acid and bile-salt stress^51,52^.

**Figure 4:**
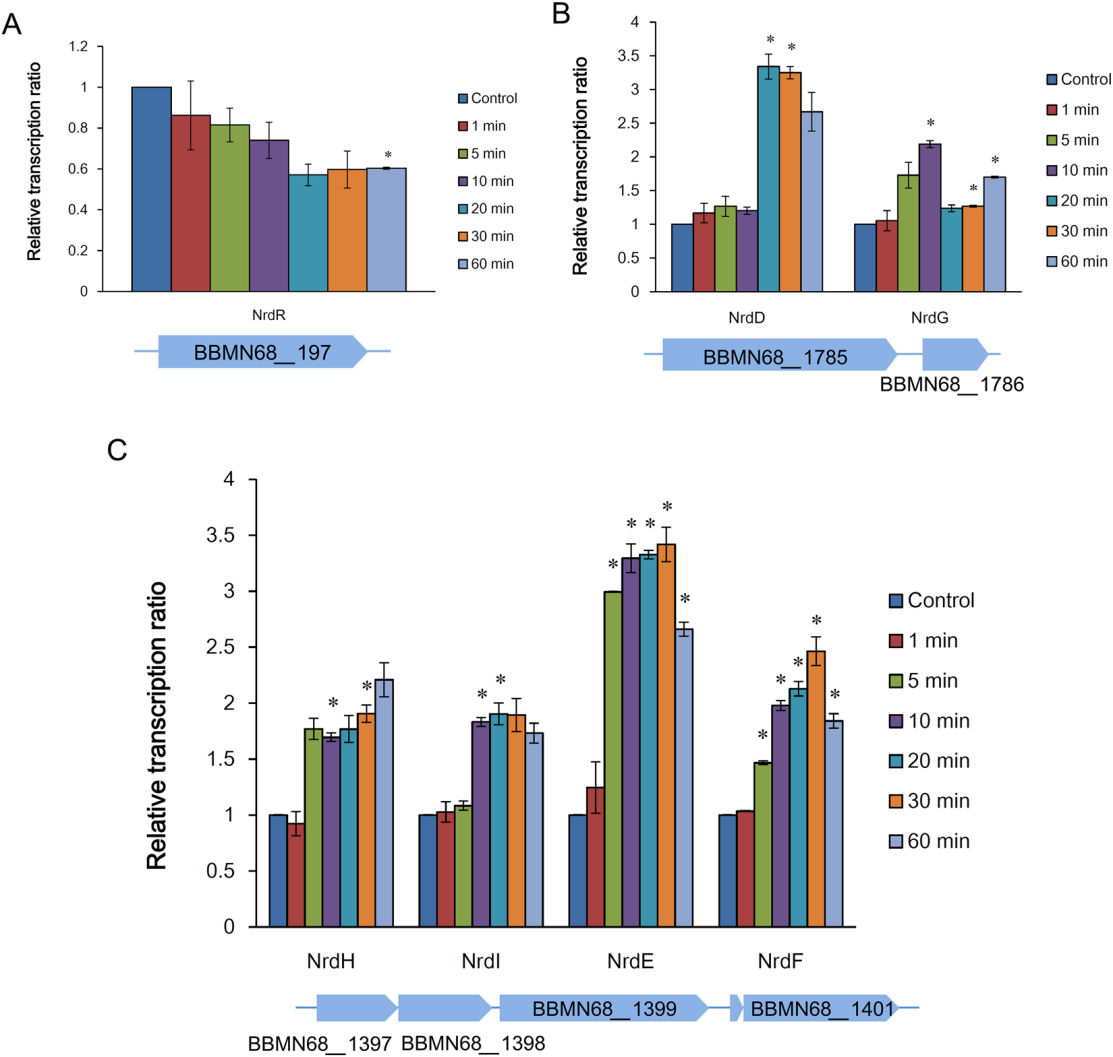
Time-course expression of ribonucleotide reductase gene clusters in *B. longum* BBMN68 response to 3% (v/v) oxygen stress detected by RT-qPCR. (**A**) Putative RNR regulator gene *nrdR* (*BBMN68_197*). (**B**) Class III RNRs *nrdDG* (*BBMN68_1785/1786*). (**C**) Class Ib RNRs *nrdHIEF* (*BBMN68_1397/1398/1399/1401*). Relative expression ratio was calculated as the ratio between signals observed in oxygen-treated samples (1 min, 5 min, 10 min, 20 min, 30 min, 60 min) and oxygen-untreated sample (Control). The mean values from three independent determinations ± SD are shown. Asterisks indicate a statistically significant difference (*P < 0.05).

The expression of ribonucleotide reductase (RNR) gene clusters, including class III RNR *nrdDG* (*BBMN68_1785/1786*), and class Ib RNR *nrdHIEF* operon (*BBMN68_1397/1398/1399/1401*), was highly induced in BBMN68 in response to oxygen (Table 2). NrdDG is an oxygen-sensitive enzyme in anaerobes that is normally expressed under microaerophilic and anaerobic conditions^53^. While the class Ib RNR, which was the highest upregulated gene cluster (*nrdHIEF* operon) in this study, has been suggested to act primarily in response to oxidative stress^54^. *nrdE* upregulation in BBMN68 in response to oxygen stress has also been confirmed at the translational level^14^. However, *nrdHIEF* induction in *B. animalis* subsp. *lactis* BL-04 and *B. longum* NCC2705 in response to sublethal levels of H_2_O_2_ was only transitory^17,18^. We therefore analyzed the temporal expression of RNR cluster genes in BBMN68 upon exposure to oxygen by RT-qPCR. The result showed that *nrdD* was induced after 20 min and *nrdG* was induced after 10 min oxygen exposure (Fig. 4). In the *nrdHIEF* cluster, *nrdH*, *nrdE*, and *nrdF* were induced after 5 min, and *nrdI* was induced after 10 min oxygen exposure (Fig. 4). Accordingly, transcription of the putative transcriptional repressor NrdR-encoding gene *BBMN68_197* decreased rapidly in BBMN68 upon exposure to oxygen (Fig. 4); putative NrdR-binding sites, as determined by Rodionov and Gelfand^55^, were located in the promoter region of *BBMN68_1785* and *BBMN68_1397*, respectively (data not shown). Upregulation of RNRs supported deoxynucleoside diphosphate/deoxynucleoside triphosphate (dNDP/dNTP) biosynthesis, which could be used for turnover and scavenging of oxidatively damaged DNA in BBMN68.

Downregulation of pyrimidine-biosynthesis genes, including members of the *pyr* gene cluster (*pyrB/I/C/F2–ubiB–pyrD1/E*; *BBMN68_534–528*), was transiently observed in response to oxygen stress in BBMN68 (Table S2). Downregulation of pyrimidine biosynthesis is a common response to different stress conditions in bifidobacteria^52,56^. In contrast, genes involved in purine metabolism were upregulated, including *BBMN68_909*, *BBMN68_1636*, and *BBMN68_591* (Table S2), leading to enhanced ATP and GTP production.

Genes belonging to COG category E (amino acid transport and metabolism) were strongly and persistently induced compared to controls (Fig. 3), indicating that the processes of amino acid and protein biosynthesis, transport and metabolism are strengthened upon BBMN68 exposure to oxygen-induced oxidative stress. However, the mRNA levels of most of the ribosomal protein-encoding genes were downregulated (Table S2). Ribosomal proteins are necessary for ribosome assembly and stability. It has been suggested that ribosomal protein synthesis is controlled primarily at the translational level, and that rRNA transcription is the rate-limiting step in ribosome synthesis in model organisms such as *E. coli* and *B. subtilis*^57^. It is speculated that the downregulation of ribosomal protein-encoding genes will have less influence on protein synthesis in BBMN68. A similar observation has been reported in *B. longum* in response to low pH and heat stress^43,45^, and in *Lactobacillus rhamnosus* in response to bile salt stress^56^. Nevertheless, most of the tRNA-encoding genes were transiently upregulated after 30 min but downregulated or unchanged after 60 min exposure to oxygen (except tRNA–Thr-encoding *BBMN68_tRNA39*, which was upregulated) (Table S2). This suggested that protein synthesis is strengthened at the early stage of oxidative stress in BBMN68, but is then suppressed at the later stage. Since the target of ROS are biomacromolecules, such as nucleic acids and proteins, their protection from ROS damage is essential for cell survival under oxidative stress^58^. From the observation that most tRNAs were first upregulated and then downregulated, together with the strong upregulation of chaperones and proteases, we hypothesized that *B. longum* BBMN68 reduces the global rates of protein synthesis, along with enhanced production of chaperones and Clp proteases to promote recycling of the misfolded and aggregated proteins under oxidative stress. Such a change in expression has also been observed in *Bacteroides fragilis* in response to oxygen exposure^59^, and in *B. longum* in response to high temperature^45^.

Notably, most genes encoding the biosynthesis of the branched-chain amino acids (BCAAs) L-Ile, L-Val, and L-Leu were significantly upregulated^49^ (Table 2, Fig. S3), including *leuABCD* (*BBMN68_1222/984/1521/1522*), *ilvC1* (*BBMN68_1262*), and *ilvE* (*BBMN68_592*). Upregulation of *leuA* expression in BBMN68 in response to oxygen-induced stress had also been confirmed at the translational level^16^. Correspondingly, genes involved in BCAA transport—*livKHMGF* (*BBMN68_1747−1751*)—were also induced after both 30 and 60 min of oxygen exposure (Table 2). Transcription of BCAA synthesis-related genes has been found to be induced in bifidobacteria under conditions of low-pH and bile-salt stress^42,43,51^. Deamination of BCAAs has been postulated as a mechanism for maintaining internal cell pH^43^, but it has not been characterized in bifidobacteria’s oxidative stress response. The upregulation of BCAA biosynthesis might provide ATP for energy metabolism and hydrophobic amino acids for protein synthesis in BBMN68 in response to oxidative stress.

Expression of genes encoding Fe–S cluster-assembly proteins, including *sufB* (*BBMN68_612*), *sufD* (*BBMN68_611*), *csdB* (*BBMN68_609*, also known as *sufS*), was upregulated in BBMN68 exposed to oxygen for 60 min (Table 2). CsdB, an IscS/Nifs homolog, plays a main role in the assembly of Fe–S clusters by mobilizing the S atom of L-Cys through cysteine desulfurase activity^60^. The SufBCD complex acts as a scaffold which donates Fe–S clusters to SufA under oxidative stress and during iron starvation in *E. coli*^61^. However, gene *BBMN68_269* encoding another Fe–S cluster assembly-related protein NifS, was not significantly induced, suggesting that only the *suf* system is induced by oxidative stress in BBMN68. Thus, to restore the necessary biochemical metabolism in response to oxidative stress, BBMN68 shows adaptable strengthening of Fe–S cluster-containing protein biosynthesis.

### Oxidative stress accelerates folate biosynthesis in BBMN68

Genes involved in tetrahydrofolate (H_4_-folate) biosynthesis were upregulated in BBMN68 after 60 min oxygen exposure^49^ (Table 2, Fig. S4). H_4_-folate serves as a donor of 1-C units involved in the biosynthesis of purines, thymidine, glycine, methionine and pantothenate. In bacteria, H_4_-folate is also required for the synthesis of formylmethionyl tRNA^fMet^, which is essential for the initiation of protein synthesis^62,63^. The induction of folate biosynthesis may contribute to repairing the DNA and protein damage caused by oxidative stress in BBMN68. In addition, the aforementioned increase in GTP production from purine metabolism supports precursors for H_4_-folate synthesis.

### BBMN68 alters cell-surface properties in response to oxidative stress

**Figure 5:**
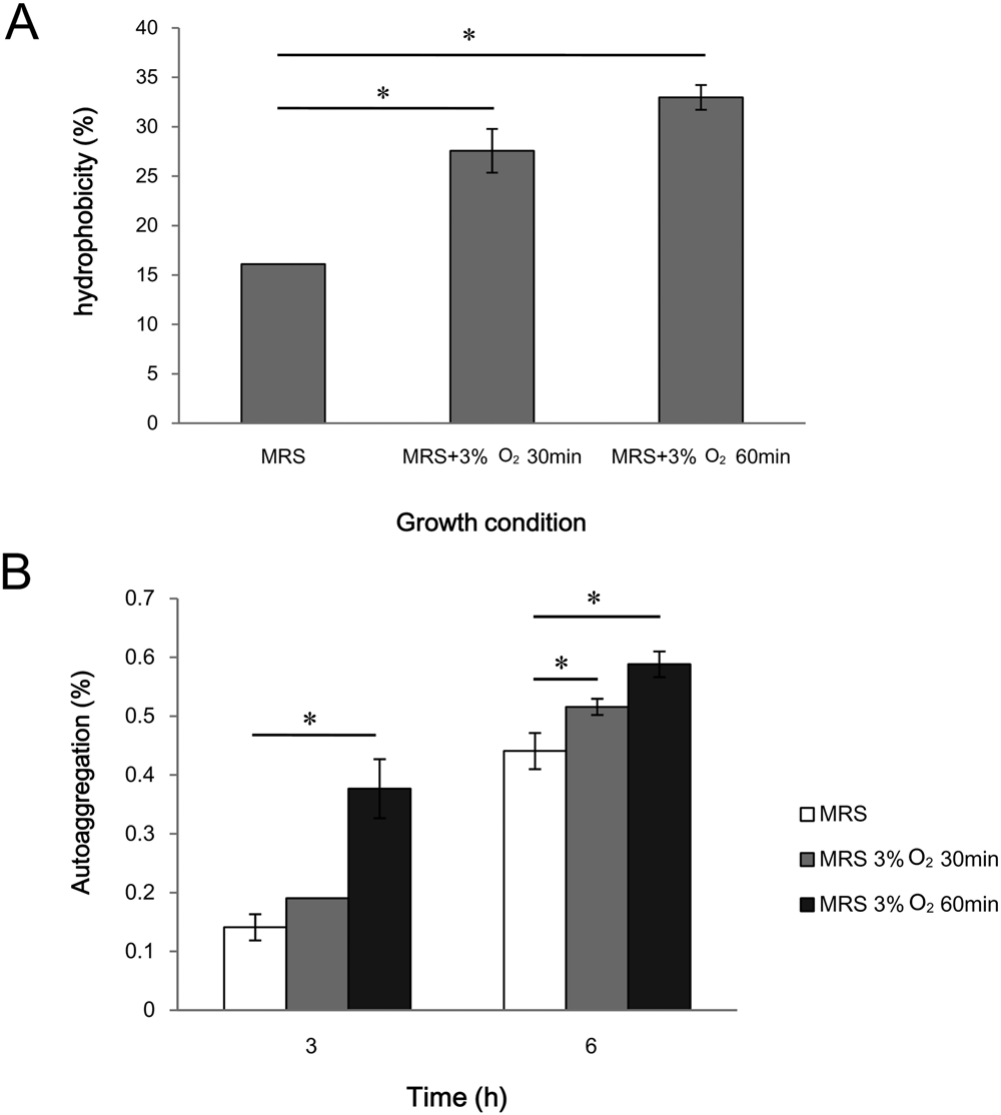
Hydrophobicity (**A**) and autoaggregation (**B**) properties of *B. longum* BBMN68 under different growth conditions. The mean values from three independent determinations ± SD are shown. Asterisks indicate a statistically significant difference (*P < 0.05).

Oxygen exposure causes changes in fatty acids in the bifidobacteria cells and an extension of the lag phase of growth; the cells become elongated and develop a rough surface due to abnormal or incomplete cell division^64^. In this study, autoaggregation and hydrophobicity properties of BBMN68 cells exposed to oxygen were increased compared to untreated cells (Fig. 5), suggesting that BBMN68 cell-surface components were modified in response to oxidative stress. Remarkably, *BBMN68_1705* encoding cyclopropane-fatty-acyl-phospholipid synthase, which catalyzes cyclopropane fatty acid biosynthesis, showed 2.71- and 4.81-fold upregulation in BBMN68 upon exposure to oxygen after 30 min and 60 min, respectively (Table 2). Cyclopropane fatty acid plays a role in the defense against environmental stresses via modification of the viscosity and permeability of cell membranes in lactic acid bacteria and bifidobacteria^51,52,65–67^. Increased cyclopropane fatty acid composition in cell membranes leads to a more hydrophobic cell surface, and the surface hydrophobicity of BBMN68 cells increased 70% to 100% upon exposure to oxygen (Fig. 5A). In addition, three adjacent operons (*BBMN68_1487–BBMN68_1490*, *BBMN68_1493–BBMN68_1491*, and *BBMN68_1494–BBMN68_1496*) encoding proteins involved in polysaccharide biosynthesis and transport were repressed in BBMN68 upon exposure to oxygen (Table 2). In particular, the transcription of two genes encoding rhamnosyltransferase was downregulated—*BBMN68_1492* and *BBMN68_1493* (Table 2). This revealed that BBMN68 reduces polysaccharide synthesis in response to oxidative stress, which might also contribute to improved hydrophobicity and autoaggregation of BBMN68 cells upon exposure to oxygen, because polysaccharides are likely to hinder cell aggregation and adhesion^68,69^. This, in turn, might reduce penetration of the surrounding dissolved oxygen into the cells^70,71^, thereby reducing the damage caused by the oxidative stress.

## Conclusion

In this study, we used RNA-Seq transcriptome profiling to investigate the mechanism governing the response to oxygen in the potentially probiotic *B. longum* strain BBMN68. Analysis of the pathways associated with the genes showing altered expression suggested that *B. longum* BBMN68 employs a complex global mechanism to cope with oxidative stress. First, the thioredoxin–thioredoxin reductase system, with thioredoxin-dependent pathways such as AhpC, provide a primary defense against ROS generated by aerobic metabolism. Moreover, several physiological processes were modulated for adaptation to the oxidative stress. To effectively cope with oxidative stress, *B. longum* BBMN68 enhanced BCAA, Fe–S, dNDP/dNTP and H_4_-folate production, toward protein and nucleotide biosynthesis and repair. In addition, *B. longum* BBMN68 increased cyclopropane-fatty-acyl-phospholipid synthase biosynthesis, while reduced cell-wall components and polysaccharide synthesis in response to oxidative stress. This could contribute to an increase in cell hydrophobicity and autoaggregation, protecting the cells from oxygen exposure. Taken together, our study provides the transcriptional landscape of *B. longum* BBMN68 grown under oxygen challenge and provides a wealth of clues for further detailed study.

## Electronic supplementary material

Supplemental information

supplementary dataset Table S2

## References

[1] Ventura M (2007). Genomics of Actinobacteria: tracing the evolutionary history of an ancient phylum. Microbiol. Mol. Biol. Rev..

[2] Lee JH, O’Sullivan DJ (2010). Genomic insights into bifidobacteria. Microbiol. Mol. Biol. Rev..

[3] Russell DA, Ross RP, Fitzgerald GF, Stanton C (2011). Metabolic activities and probiotic potential of bifidobacteria. Int. J. Food Microbiol..

[4] Shah NP (2000). Probiotic bacteria: selective enumeration and survival in dairy foods. J. Dairy Sci..

[5] Boylston TD, Vinderola CG, Ghoddusi HB, Reinheimer JA (2004). Incorporation of bifidobacteria into cheeses: challenges and rewards. Int. Dairy J..

[6] Talwalkar A, Kailasapathy K (2003). Metabolic and biochemical responses of probiotic bacteria to oxygen. J. Dairy Sci..

[7] Simpson PJ, Stanton C, Fitzgerald GF, Ross RP (2005). Intrinsic tolerance of *Bifidobacterium* species to heat and oxygen and survival following spray drying and storage. J. Appl. Microbiol..

[8] Imlay JA (2008). Cellular defenses against superoxide and hydrogen peroxide. Annu. Rev. Biochem..

[9] Brioukhanov AL, Netrusov AI (2004). Catalase and superoxide dismutase: distribution, properties, and physiological role in cells of strict anaerobes. Biochemistry-Moscow.

[10] Brioukhanov AL, Netrusov A (2007). Aerotolerance of strictly anaerobic microorganisms and factors of defense against oxidative stress: A review. Appl. Biochem. Microbiol..

[11] O’Callaghan A, van Sinderen D (2016). Bifidobacteria and their role as members of the human gut microbiota. Front. Microbiol..

[12] Hayashi K (2013). Purification and characterization of oxygen-inducible haem catalase from oxygen-tolerant *Bifidobacterium asteroides*. Microbiology.

[13] Zuo FL (2014). Homologous overexpression of alkyl hydroperoxide reductase subunit C (*ahpC*) protects *Bifidobacterium longum* strain NCC2705 from oxidative stress. Res. Microbiol..

[14] Ruiz L (2012). Molecular clues to understand the aerotolerance phenotype of *Bifidobacterium animalis* subsp. *lactis*. Appl. Environ. Microbiol..

[15] Schell MA (2002). The genome sequence of *Bifidobacterium longum* reflects its adaptation to the human gastrointestinal tract. Proc. Natl. Acad. Sci. USA.

[16] Xiao M (2011). Oxidative stress-related responses of *Bifidobacterium longum* subsp. *longum* BBMN68 at the proteomic level after exposure to oxygen. Microbiology..

[17] Oberg TS, Ward RE, Steele JL, Broadbent JR (2013). Genetic and physiological responses of *Bifidobacterium animalis* subsp. *lactis* to hydrogen peroxide stress. J. Bacteriol..

[18] Oberg TS, Ward RE, Steele JL, Broadbent JR (2015). Transcriptome analysis of *Bifidobacterium longum* strains that show a differential response to hydrogen peroxide stress. J. Biotechnol..

[19] Zomer A, van Sinderen D (2010). Intertwinement of stress response regulons in *Bifidobacterium breve* UCC2003. Gut Microbes.

[20] Zomer A (2010). An interactive regulatory network controls stress response in *Bifidobacterium breve* UCC2003. J. Bacteriol..

[21] Erill I, Campoy S, Barbé J (2007). Aeons of distress: an evolutionary perspective on the bacterial SOS response. FEMS Microbiol. Rev..

[22] Berger B, Moine D, Mansourian R, Arigoni F (2010). HspR mutations are naturally selected in *Bifidobacterium longum* when successive heat shock treatments are applied. J. Bacteriol..

[23] Shimamura S (1992). Relationship between oxygen sensitivity and oxygen metabolism of *Bifidobacterium* species. J. Dairy Sci..

[24] Yang HY (2009). Oral administration of live *Bifidobacterium* substrains isolated from centenarians enhances intestinal function in mice. Curr. Microbiol..

[25] Yang HY (2009). Oral administration of live *Bifidobacterium* substrains isolated from healthy centenarians enhanced immune function in BALB/c mice. Nutr. Res..

[26] Yang J (2015). *Bifidobacterium longum* BBMN68-specific modulated dendritic cells alleviate allergic responses to bovine β-lactoglobulin in mice. J. Appl. Microbiol..

[27] Hao YL (2011). Complete Genome Sequence of *Bifidobacterium longum* subsp. *longum* BBMN68, a New Strain from a Healthy Chinese Centenarian. J. Bacteriol..

[28] Schmittgen TD, Livak KJ (2008). Analyzing real-time PCR data by the comparative CT method. Nat. Protoc..

[29] Del Re B, Dgorbati B, Miglioli M, Palenzona D (2000). Adhesion, autoaggregation and hydrophobicity of 13 strains of *Bifidobacterium longum*. Lett. Appl. Microbiol..

[30] Pablo FP, Yessica M, Edgardo AD, Graciela LDA (1998). Surface properties of bifidobacterial strains of human origin. Appl. Environ. Microbiol..

[31] Pan WH, Li PL, Liu ZY (2006). The correlation between surface hydrophobicity and adherence of *Bifidobacterium* strains from centenarians’ faeces. Anaerobe.

[32] Tatusov RL, Koonin EV, Lipman DJ (1997). A genomic perspective on protein families. Science.

[33] Carmel-Harel O, Storz G (2000). Roles of the glutathione-and thioredoxin-dependent reduction systems in the *Escherichia coli* and *Saccharomyces cerevisiae* responses to oxidative stress. Ann. Rev. Microbiol..

[34] Jordan A, Åslund F, Pontis E, Reichard P, Holmgren A (1997). Characterization of *Escherichia coli* NrdH: a glutaredoxin-like protein with a thioredoxin-like activity profile. J. Biol. Chem..

[35] Zeller T, Klug G (2006). Thioredoxins in bacteria: functions in oxidative stress response and regulation of thioredoxin genes. Naturwissenschaften.

[36] Zuo FL (2014). Combination of heterogeneous catalase and superoxide dismutase protects *Bifidobacterium longum* strain NCC2705 from oxidative stress. Appl. Microbiol. Biotechnol..

[37] Cortial S (2010). NADH oxidase activity of *Bacillus subtilis* nitroreductase NfrA1: insight into its biological role. FEBS Lett..

[38] Mérmod M (2010). Structure and function of CinD (YtjD) of *Lactococcus lactis*, a copper-induced nitroreductase involved in defense against oxidative stress. J. Bacteriol..

[39] Streker K, Freiberg C, Labischinski H, Hacker J, Ohlsen K (2005). *Staphylococcus aureus* NfrA (SA0367) is a flavin mononucleotide-dependent NADPH oxidase involved in oxidative stress response. J. Bacteriol..

[40] Horsburgh MJ, Wharton SJ, Karavolos M, Foster SJ (2002). Manganese: elemental defence for a life with oxygen. Trends Microbiol..

[41] Klijn A, Mercenier A, Arigoni F (2005). Lessons from the genomes of bifidobacteria. FEMS Microbiol. Rev..

[42] Sánchez B (2005). Proteomic analysis of global changes in protein expression during bile salt exposure of *Bifidobacterium longum* NCIMB 8809. J. Bacteriol..

[43] Sánchez B (2007). Low-pH adaptation and the acid tolerance response of *Bifidobacterium longum* biotype *longum*. Appl. Environ. Microbiol..

[44] Savijoki K (2005). Effect of heat-shock and bile salts on protein synthesis of *Bifidobacterium longum* revealed by [35S]methionine labelling and two dimensional gel electrophoresis. FEMS Microbiol. Lett..

[45] Rezzonico E (2007). Global transcriptome analysis of the heat shock response of *Bifidobacterium longum*. FEMS Microbiol. Lett..

[46] Zolkiewski M (1999). ClpB cooperates with DnaK, DnaJ, and GrpE in suppressing protein aggregation: A novel multi-chaperone system from *Escherichia coli*. J. Biol. Chem..

[47] Lund PA (2001). Microbial molecular chaperones. Adv. Microb. Physiol..

[48] Stohl EA, Criss AK, Seifert HS (2005). The transcriptome response of *Neisseria gonorrhoeae* to hydrogen peroxide reveals genes with previously uncharacterized roles in oxidative damage protection. Mol. Microbiol..

[49] Kanehisa M, Goto S (2000). KEGG: Kyoto Encyclopedia of Genes and Genomes. Nucleic Acids Res..

[50] Parche S (2007). Sugar transport systems of *Bifidobacterium longum* NCC2705. J. Mol. Microbiol. Biotechnol..

[51] Jin JH (2012). Mechanism analysis of acid tolerance response of *Bifidobacterium longum* subsp. *longum* BBMN68 by gene expression profile using RNA-Sequencing. PLOS ONE.

[52] An HR (2014). Integrated transcriptomic and proteomic analysis of the bile stress response in a centenarian-originated probiotic *Bifidobacterium longum* BBMN68. Mol. Cell Proteomics.

[53] Torrents E (2007). NrdR controls differential expression of the *Escherichia coli* ribonucleotide reductase genes. J. Bacteriol..

[54] Monje-Casas F, Jurado J, Prieto-Alamo MJ, Holmgren A, Pueyo C (2001). Expression analysis of the *nrdHIEF* operon from *Escherichia coli*. Conditions that trigger the transcript level *in vivo*. J. Biol. Chem..

[55] Rodionov DA, Gelfand MS (2005). Identification of a bacterial regulatory system for ribonucleotide reductases by phylogenetic profiling. Trends Genet..

[56] Koskenniemi K (2011). Proteomics and transcriptomics characterization of bile stress responsein probiotic *Lactobacillus rhamnosus* GG. Mol. Cell. Proteomics.

[57] Paul BJ, Ross W, Gaal T, Gourse RL (2004). rRNA transcription in *Escherichia coli*. Annu. Rev. Genet..

[58] Imlay JA (2013). The molecular mechanisms and physiological consequences of oxidative stress: lessons from a model bacterium. Nat. Rev. Microbiol..

[59] Sund CJ (2008). The *Bacteroides fragilis* transcriptome response to oxygen and H_2_O_2_: the role of OxyR and its effect on survival and virulence. Mol. Microbiol..

[60] Kurihara T, Mihara H, Kato S, Yoshimura T, Esaki N (2003). Assembly of iron-sulfur clusters mediated by cysteine desulfurases, IscS, CsdB and CSD, from *Escherichia coli*. BBA-Proteins Proteom..

[61] Py B, Moreau PL, Barras F (2011). Fe-S clusters, fragile sentinels of the cell. Curr. Opin. Microbiol..

[62] Bermingham A (2002). & Derrick, J. P. The folic acid biosynthesis pathway in bacteria: evaluation of potential for antibacterial drug discovery. BioEssays.

[63] Levin I, Giladi M, Altman-Price N, Ortenberg R, Mevarech M (2004). An alternative pathway for reduced folate biosynthesis in bacteria and halophilic archaea. Mol. Microbiol..

[64] Ahn JB, Hwang HJ, Park JH (2001). Physiological responses of oxygen-tolerant anaerobic *Bifidobacterium longum* under oxygen. J. Microbiol. Biotechnol..

[65] Guillot A, Obis D, Mistou MY (2000). Fatty acid membrane composition and activation of glycine-betaine transport in *Lactococcus lactis* subjected to osmotic stress. Int. J. Food Microbiol..

[66] Grandvalet C (2008). Changes in membrane lipid composition in ethanol- and acid-adapted *Oenococcus oeni* cells: characterization of the cfa gene by heterologous complementation. Microbiology.

[67] Montanari C, Sado Kamdem SL, Serrazanetti DI, Etoa FX, Guerzoni ME (2010). Synthesis of cyclopropane fatty acids in *Lactobacillus helveticus* and *Lactobacillus sanfranciscensis* and their cellular fatty acids changes following short term acid and cold stresses. Food Microbiol..

[68] Aires J (2010). Proteomic comparison of the cytosolic proteins of three *Bifidobacterium longum* human isolates and *B. longum* NCC2705. BMC Microbiol..

[69] Polak-Brecka M, Waśko A, Paduch R, Skrzypek T, Sroka-Bartnicka A (2014). The effect of cell surface components on adhesion ability of *Lactobacillus rhamnosus*. Antonie van Leeuwenhoek.

[70] Sigalevich P, Meshorer E, Helman Y, Cohen Y (2000). Transition from anaerobic to aerobic growth conditions for the sulfate-reducing bacterium *Desulfovibrio oxyclinae* results in flocculation. Appl. Environ. Microbiol..

[71] McLean JS (2008). Oxygen-dependent autoaggregation in *Shewanella oneidensis* MR-1. Environ. Microbiol..

