# Supplementary material for: Transcriptomic analysis of Bifidobacterium longum subsp. longum BBMN68 in response to oxidative shock
Source: Sci Rep. 2018 Nov 20;8:17085. doi: 10.1038/s41598-018-35286-7 (PMC6244367; doi:10.1038/s41598-018-35286-7)
Supplement: Supplementary file 1 — Supplemental information [file 41598_2018_35286_MOESM1_ESM.pdf]

Supplemental information

Transcriptomic analysis of *Bifidobacterium longum* subsp. *longum* BBMN68 in response to oxidative shock

Fanglei Zuo[a,b,†], Rui Yu[a,b], Man Xiao[a,b], Gul Bahar Khaskheli[a,b], Xiaofei Sun[a], Huiqin Ma[c], Fazheng Ren[a], Bing Zhang[d], Shangwu Chen[a,b,*]

[a] *Beijing Advanced Innovation Center for Food Nutrition and Human Health, College of Food Science and Nutritional Engineering, China Agricultural University, Beijing 100083, P. R. China*

[b] *Department of Food Science and Engineering, College of Food Science and Nutritional Engineering, China Agricultural University, Beijing 100083, P. R. China*

[c] *College of Horticulture, China Agricultural University, Beijing 100193, P. R. China*

[d] *Core Genomic Facility, Beijing Institute of Genomics, Chinese Academy of Sciences, Beijing 100101, P. R. China*

[†] *Present address: Fanglei Zuo, Department of Molecular Biosciences, The Wenner-Gren Institute, Stockholm University, SE-10691 Stockholm, Sweden*

[*] *Corresponding author, swchen@cau.edu.cn*

Supplementary Table S1. Fold change in expression of up- and downregulated genes of *B. longum* BBMN68 under oxygen treatment.

Changes in mRNA level (30 min vs. control)	Number of genes (% of total) with indicated mRNA level change			
	FC ≥ 2	2 ≤ FC < 5	5 ≤ FC < 10	FC ≥ 10
Upregulated	241 (13.16)	206 (11.25)	23 (1.26)	12 (0.66)
Downregulated	99 (5.41)	94 (5.13)	5 (0.27)	0 (0)
Total	340 (18.57)	300 (16.38)	28 (1.53)	12 (0.66)
Changes in mRNA level (60 min vs. control)	Number of genes (% of total) with indicated mRNA level change			
	FC ≥ 2	2 ≤ FC < 5	5 ≤ FC < 10	FC ≥ 10
Upregulated	217 (11.84)	193 (10.53)	19 (1.04)	5 (0.27)
Downregulated	218 (11.90)	202 (11.03)	12 (0.66)	4 (0.22)
Total	435 (23.74)	395 (21.56)	31 (0.17)	9 (0.49)

FC: fold change.

Supplementary Table S2. Genes that were upregulated or downregulated by ≥2-fold in the transcriptome of *B. longum* BBMN68 exposed to 3% (v/v) oxygen (see Excel file).

Supplementary Figure S1. Growth of *B. longum* BBMN68 under modified MRS broth supplemented with $MnSO_4$ (at 0 to 1000 μM). *B. longum* BBMN68 overnight culture was inoculated at 1% into an injection vial containing modified MRS broth and grown upon exposure to 3% (v/v) oxygen (A) or under anaerobic conditions (B).

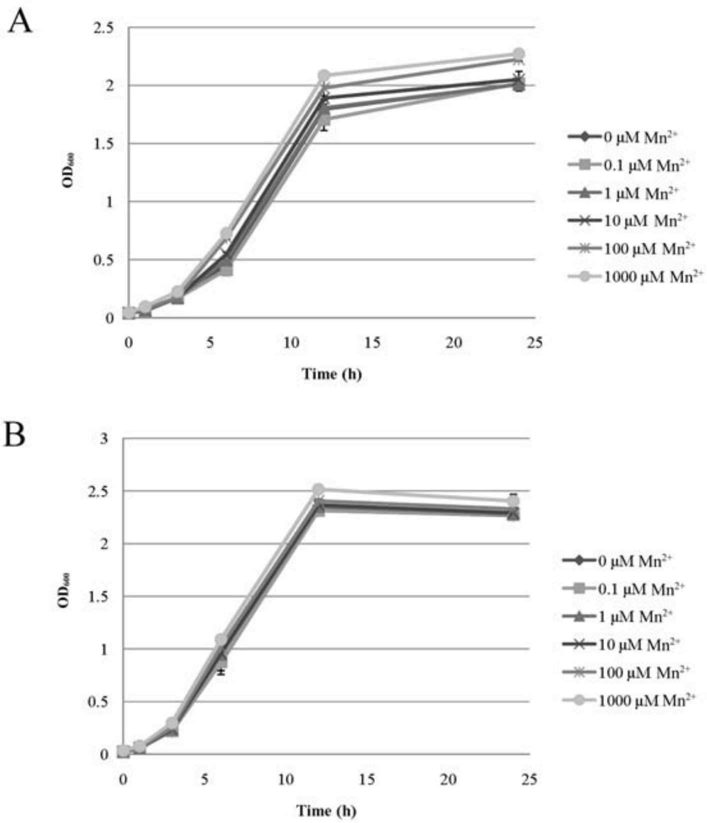

Supplementary Figure S2. Global transcriptome profile of carbohydrate metabolism (adapted from KEGG pathway blb00010, blb00020, blb00030, and blb00052)[49] of *B. longum* BBMN68 exposed to 3% (v/v) oxygen. The gene designation and enzyme code are shown in and adjacent to the boxes, respectively. The small colored squares adjacent to genes indicate increased expression fold change for 3% oxygen for 30 min treatment vs. control (left), and 3% oxygen for 60 min treatment vs. control (right). Square colors indicate the extent of the fold changes according to color key at bottom left.

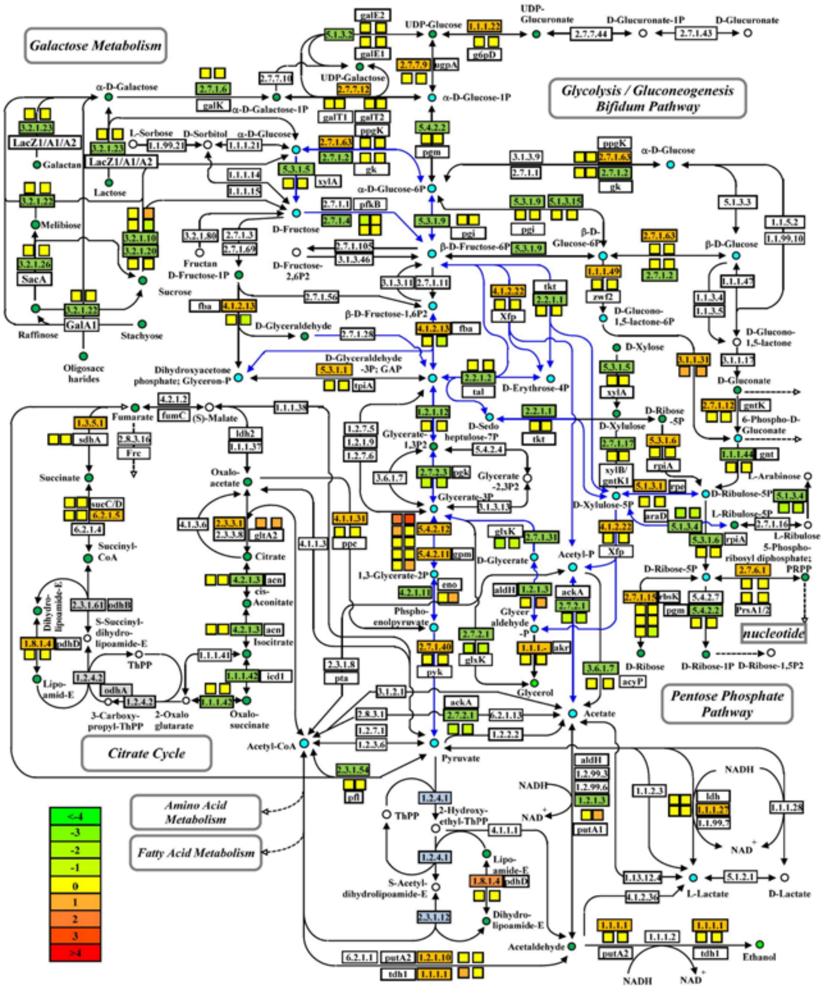

Supplementary Figure S3. Genes involved in branched-chain amino acid (BCAA) synthesis (adapted from KEGG pathway blb00290)[49] were upregulated in *B. longum* BBMN68 upon exposure to 3% (v/v) oxygen. Gene name and enzyme code are shown. The small colored squares adjacent to genes indicate increased (red) or non-significantly increased (yellow) mRNA expression for 3% oxygen treatment for 30 min vs. control (left), and 3% oxygen treatment for 60 min vs. control (right).

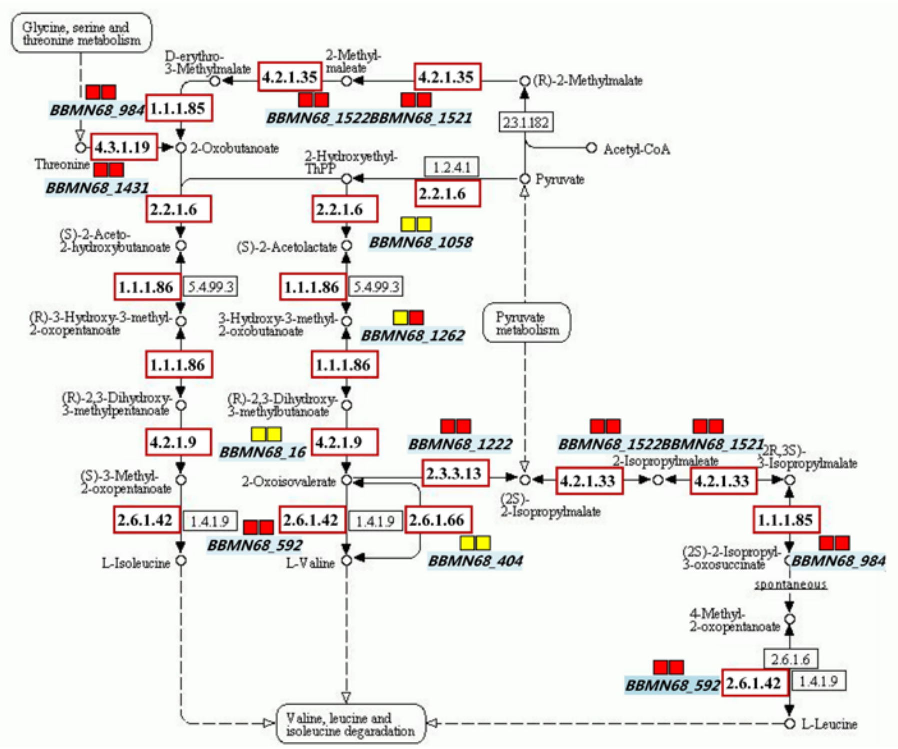

Supplementary Figure S4. Genes involved in tetrahydrofolate (THF) synthesis (adapted from KEGG pathway blb00790)[49] were upregulated in *B. longum* BBMN68 upon exposure to 3% (v/v) oxygen. Red gene names indicate upregulation in BBMN68 exposed to 3% oxygen for 60 min vs. control.

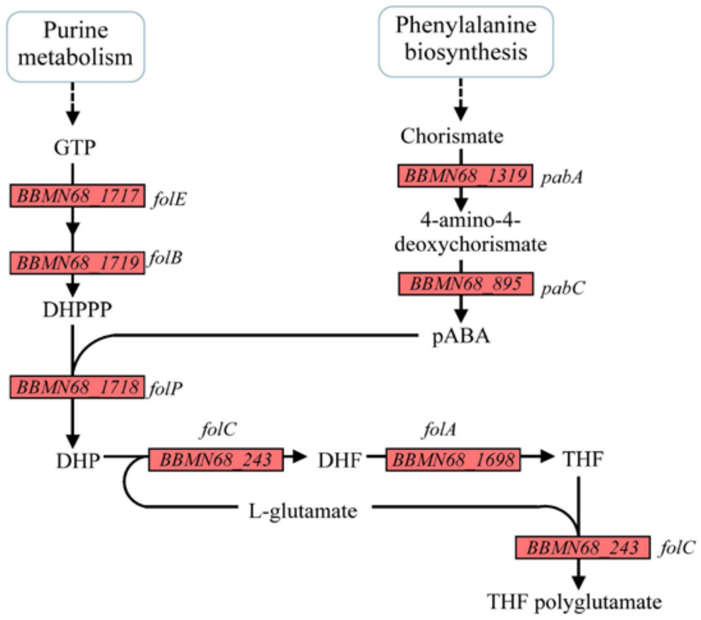

Reference

49. Kanehisa, M. & Goto, S. KEGG: Kyoto Encyclopedia of Genes and Genomes. *Nucleic Acids Res*. **28**, 27-30 (2000).
